# Traditional Chinese herbal bath therapy for insomnia: A protocol for systematic review

**DOI:** 10.1097/MD.0000000000021166

**Published:** 2020-07-17

**Authors:** Qiao Wen, Xiaohui Dong, Lei Lan, Lizhou Liu, Steve Tumilty, Bohua Yan, Yabing Chen, Xing Wang, Yonggang Zhang, Fen Feng

**Affiliations:** a Acupuncture and Tuina School, The 3rd Teaching Hospital, Chengdu University of Traditional Chinese Medicine, Chengdu, China; b Centre for Health, Activity, and Rehabilitation Research, School of Physiotherapy, University of Otago, Dunedin, New Zealand; c Hospital of Chengdu University of Traditional Chinese Medicine, Chengdu; d Santai County Hospital of Traditional Chinese Medicine, Santai County; e Department of Periodical Press and National Clinical Research Center for Geriatrics, West China Hospital, Sichuan University, Chengdu, China.

**Keywords:** baths, insomnia, protocol, systematic review, traditional Chinese medicine

## Abstract

**Introduction:**

Insomnia is a major public health problem. Due to the side effects of pharmacological therapy, people are seeking to choose complementary and alternative therapies for insomnia disorder. Traditional Chinese herbal bath therapy is an important complementary therapy which combines advantages of Chinese herbs and bathing therapy. This protocol describes the methodology of a systematic review assessing the effectiveness and safety of traditional Chinese herbal bath therapy for insomnia.

**Methods and analysis:**

Reporting of this review will be adherent to the Preferred Reporting Items for Systematic Reviews and Meta-Analyses (PRISMA) statement. We will electronically search the following seven databases from inception to January 23, 2020: PubMed, Cochrane database (CENTRAL), EMBASE, China National Knowledge Infrastructure (CNKI), Chinese Biomedical Literature Database (CBM), VIP Database, and Wanfang Database. Parallel randomized controlled trials evaluating the effectiveness and safety of traditional Chinese herbal bath therapy for insomnia will be included. Study selection, data extraction and assessment of risk of bias will be performed independently by two researchers. The sleep quality will be assessed as the primary outcome. Global symptom improvement, anxiety and depression, and adverse events will be evaluated as secondary outcomes. The Cochrane's risk of bias tool will be utilized for assessing the methodological quality of included studies. Revman software (v.5.3) will be used for data synthesis and statistical analysis. Data will be synthesized by either fixed-effects or random-effects model according to a heterogeneity test. If it is not appropriate for a meta-analysis, a descriptive analysis will be conducted. GRADE system will be used to assess the quality of evidence.

**PROSPERO registration number:**

CRD42020168507

## Abstract 2

Supplemental Digital Content is available in the text

## 1 Introduction

Insomnia is the most common mental disorders.^[1]^ It is characterized by difficulties initiating or maintaining sleep, or early morning awakening associated with impaired daytime functioning.^[2]^ The National Institute of Health defines insomnia disorder as “dysregulation of sleep homeostasis, sleep deficiency, sleep fragmentation, insufficient sleep or impairment of sleep quality or quantity caused by a sleep disorder.”^[3]^ Insufficient sleep will not only affect the quality of life of the patients but also affect psychological and physical health and may cause other conditions, such as anxiety, depression, fatigue, and decreased cognitive function.^[4–6]^ The prevalence of sleep disturbance ranges between 36% and 50%,^[7,8]^ and over 6.5 million Americans suffer from chronic insomnia.^[9]^ Nearly 15% of the population in China meets the criteria for insomnia.^[10]^ The prevalence of insomnia imposes a high economic burden to the individuals and society.^[11–14]^

At present, treatments for insomnia disorder mainly include pharmacological and non-pharmacological therapies.^[15]^ Hypnotics are currently the most commonly used pharmacological therapy. However, long-term use of hypnotics is not generally recommended due to lack of evidence and side effects.^[2]^ Side effects of hypnotics such as tolerance, dependency, cognitive function impairment, and daytime sedation and confusion may result in poor compliance, drug abuse, and diminished quality of life.^[15,16]^ Non-pharmacological therapies have low risk of drug-related side effects. Thus, an increasing number of patients turn to non-pharmacological therapies to improve sleeping condition. Cognitive behavioral therapy for insomnia (CBT-I), though being a standard of non-pharmacological therapy for insomnia, is underutilized because of a scarcity of CBT-I providers, limited insurance coverage, non-responsiveness, and poor compliance.^[2,17,18]^ Therefore, new complementary and alternative therapies with favorable benefit to risk ratio and availability are potential options for insomnia suffers.

Traditional Chinese herbal bath therapy, combining balneotherapy with specific Chinese herbs, has been used over thousands of years in China. Due to its wide availability, usability and high compliance, it is recommended as a simple and convenient external therapy for managing insomnia. Based upon the basic theory of traditional Chinese medicine, active ingredients of Chinese herbs are absorbed through the skin acting on the meridians and acupoints to play a therapeutic role. Studies have proven that foot baths with warm water can promote sleep and improve sleep quality by increasing limb blood perfusion to regulate body temperature.^[19–21]^ The previous systematic review has shown that Chinese herbal medicine can increase insomnia patients’ sleep quality and improve their psychological health,^[22]^ and the sedative-hypnotic functions of Chinese herbal medicine are mediated by the GABAergic system.^[23,24]^ It may be reasonable to predict that the combination of bath therapy and Chinese herbal medicine is effective for patients with insomnia. However, the strength of evidence for the effects of traditional Chinese herbal bath therapy on sleep quality is unknown. Therefore, the goal of this article is to assess the effectiveness of traditional Chinese herbal bath therapy for unsatisfied sleep quality of insomnia patients.

## 2 Objectives

To assess the effectiveness and safety of traditional Chinese herbal bath therapy for insomnia disorder.

## 3 Methods

This systematic review has been registered with the International Prospective Register of Systematic Reviews (PROSPERO) (registration number: CRD42020168507). Reporting of this review protocol has been adhering to the Preferred Reporting Items for Systematic Reviews and Meta-Analyses Protocols (PRISMA-P) statement.^[25]^ The PRISMA-P checklist is provided in Appendix A.

### 3.1 Types of studies

Parallel randomized controlled trials (RCTs) that evaluated the effectiveness and safety of traditional Chinese herbal bath therapy for managing insomnia disorder will be included in this systematic review. No restrictions will be made on publication status, language or year of publication. Cohort studies, case series, case reports, reviews, animal mechanism studies, non-RCTs, and RCT protocols will be excluded.

### 3.2 Types of participants

Participants with a diagnosis of insomnia will be included. Diagnosis criteria include the Diagnostic and Statistical Manual of Mental Disorders (DSM-IV, DSM-5),^[26,27]^ International Classification of Sleep Disorders (ICSD-3),^[28]^ International Statistical Classification of Diseases and Health-Related Problems (ICD-10),^[29]^ and Chinese Classification of Mental Disorders (CCMD-3).^[30]^ No restrictions will be applied to age, gender, ethnicity, and source of cases.

### 3.3 Types of interventions

Studies will be included if they aimed to improve sleeping through traditional Chinese herbal bath therapy alone or in combination with hypnotics or standard care. Studies involving traditional Chinese herbal bath therapy combined with other therapies will be included if the other therapies were used equally in both the experimental and the control groups. Studies comparing different types, duration or frequency of traditional Chinese herbal bath therapy will be excluded.

### 3.4 Types of comparators

1. Traditional Chinese herbal bath therapy versus warm water bath therapy;
2. Traditional Chinese herbal bath therapy alone or in combination with hypnotics versus hypnotics;
3. Traditional Chinese herbal bath therapy alone or in combination with standard care versus standard care;
4. Traditional Chinese herbal bath therapy in combination with other therapies versus other therapies.

### 3.5 Types of outcome measures

#### 3.5.1 Primary outcomes

The primary outcomes are the sleep quality assessed by the Pittsburgh Sleep Quality Index (PSQI),^[31]^ Insomnia Severity Index (ISI),^[32]^ and Spiegel Sleep Questionnaire (SSQ).

#### 3.5.2 Secondary outcomes

1. Global symptom improvement, which is measured by scales such as effective rate and traditional Chinese medicine symptom scores^[33]^;
2. Effect on anxiety and depression, which is measured by scales such as the Hamilton Anxiety Rating Scale (HAM-A),^[34]^ Self-rating Anxiety Scale (SAS),^[35]^ and Self-rating Depression Scale (SDS)^[36]^;
3. Adverse events.

### 3.6 Timing of outcome assessment

Studies in which the primary outcomes were reported at pre-intervention and at least one post-intervention time point are eligible for inclusion. The follow-up measurements reported at the latest time point will be extracted for analysis.

### 3.7 Information sources and search strategy

The search will be performed in English and Chinese. The following seven databases will be searched from inception to January 23, 2020: PubMed, Cochrane database (CENTRAL), EMBASE, China National Knowledge Infrastructure (CNKI), Chinese Biomedical Literature Database (CBM), VIP Database, and Wanfang Database.

The CENTRAL search strategy is shown in Table 1. The search strategy is based on previous review articles,^[37–39]^ and the search words will be modified for Chinese medical terms in the four Chinese databases.

**Table 1:**
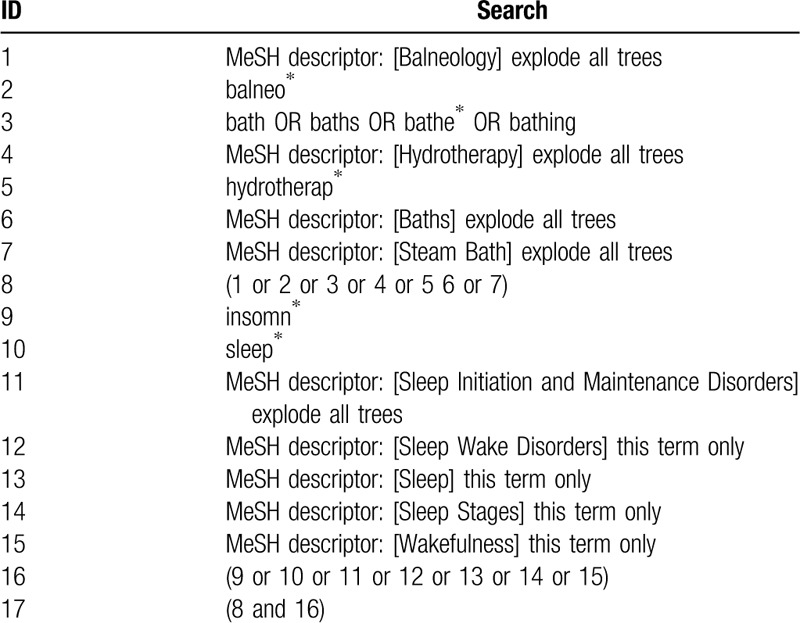
Search strategy used in Cochrane database.

The WHO International Clinical Trials Registry Platform (ICTRP), Chinese Clinical Trial Register, and ClinicalTrials.gov will be searched using the term “bath” and “sleep” to identify unpublished and ongoing studies.

### 3.8 Searching other resources

To augment the results of the database search, the bibliographies of identified studies, relevant reports, and reviews will be manually searched. We will also contact the relevant experts and organizations for information about unpublished and ongoing studies.

### 3.9 Selection of studies

Two investigators (WQ, DX) will independently search, and assess study eligibility based on their titles and abstracts after removing duplicates. Full texts of the potential studies will be obtained for further inspection. If we identify multiple reports of the same study, we will link them together. We will contact the corresponding authors for additional information if needed. Any disagreements will be addressed by discussion, consulting a third author (FF) if necessary. Reasons for exclusions will be independently identified and recorded. The selection process of study identification and its results will be outlined as a PRISMA flow diagram.

### 3.10 Data extraction and management

We have developed a pre-specified data collection form and performed a pretest, which will include study design and setting, participant characteristics (e.g., demographics, diagnostic criteria, comorbidity), interventions and comparators (e.g., the composition of the herbal bath formula, the type of control, duration and frequency of treatment), outcomes, and adverse events. Two investigators (WQ, DX) will independently complete the data collection form for all eligible studies. The corresponding authors will be contacted to request insufficient or missing information. We will resolve disagreements by discussion or by appeal to a third author (FF). The data will be stored using Microsoft Excel.

### 3.11 Assessment of risk of bias in included studies

Two investigators (WQ, DX) will independently appraise the risk of bias for each study and cross-check the results. The following domains will be assessed in accordance with the Cochrane's tool for assessing risk of bias^[40]^:

1. bias arising from the randomization process,
2. bias due to deviations from intended interventions,
3. bias due to missing outcome data,
4. bias in measurement of the outcome, and
5. bias in selection of the reported result.

Protocols and registration information of the studies will be used to assess the risk of selective reporting bias. The results of the assessment including the verbatim quotes and rationale for each study will be presented in the risk of bias table. We will assign low risk of bias, some concerns, or high risk of bias to each domain. Discrepancies in particular studies will be resolved by discussion or by appeal to a third author (FF).

### 3.12 Data synthesis

Review Manager V5.3 will be utilized for data synthesis and statistical analysis. Dichotomous data will be presented using relative risk (RR) with 95% confidence interval (95% CI). Adverse events and cases where there are no events in either group will be presented using risk difference (RD). Continuous outcomes will be presented using mean differences (MDs) for all outcomes measured on the same scale and the standardized mean differences (SMDs) for all outcomes measured on different validated scales together with 95% CI.

Heterogeneity will be assessed by visual inspection of forest plots and using the Chi^2^ test as well as the *I*^2^ statistic. The suggested thresholds for interpretation of *I*^2^ percentages are as follows:

1. might not be important (0–40%),
2. may represent moderate heterogeneity (30–60%),
3. may represent substantial heterogeneity (50–90%), and
4. considerable heterogeneity (75–100%).^[41]^

The random-effects model will be applied for the pooled data if the statistical heterogeneity exists (*P* < .1 and *I*^2^ ≥ 50%). Otherwise, the fixed-effects model using the Mantel–Haenszel method will be applied. We will investigate the possible reasons for the heterogeneity by checking the data and performing subgroup analysis. If there is substantial heterogeneity, meta-analysis is considered inappropriate. A narrative synthesis will be presented to summarise and describe the characteristics and findings of the included studies.

### 3.13 Sensitivity analysis

Sensitivity analysis will be conducted to determine the robustness of our conclusions. The analysis will investigate the potential impact of the missing data, statistical model (random-effects models versus fixed-effect models), and risk of bias levels.

### 3.14 Subgroup analysis

In the presence of sufficient data, we will carry out the following subgroup analyses:

1. Primary insomnia versus secondary insomnia;
2. Adults aged below 65 versus adults aged over 65;
3. Short-term treatment (≤4 weeks) vs long-term treatment (>4 weeks).

### 3.15 Assessment of reporting biases

Funnel plots will be used to explore the publication bias when 10 or more trials are included.

### 3.16 Summary of evidence

Two investigators (WQ, DX) will assess the quality of evidence for all outcomes independently in accordance with the Grading of Recommendations Assessment, Development and Evaluation (GRADE)^[42]^ guidelines, and present the results in the “summary of findings” table. The five considerations of GRADE include risk of bias, consistency of effect, imprecision, indirectness, and publication bias. Evidence quality will be rated into four levels: high, moderate, low, or very low.

### 3.17 Patient and public involvement

Patient and public were not involved.

## 4 Discussion

With the growing public concerns about sleep quality and increasing interest in complementary and alternative therapies, an increasing number of people seek complementary and alternative therapies for the treatment of insomnia. The traditional Chinese herbal bath therapy, as an important complementary treatment, is wildly used in China as a low-cost and convenient means of managing insomnia. It is likely to have fewer harms, whereas pharmacological therapy can be associated with serious side effects. Considering a number of randomized controlled trials on traditional Chinese herbal bath therapy have been published in recent years, a critical evaluation and comprehensive synthesis of the available evidence of traditional Chinese herbal bath therapy is warranted.

To the best of our knowledge, this is the first systematic review to investigate the clinical effectiveness and safety of traditional Chinese herbal bath therapy for insomnia disorder. Meanwhile, the composition of the commonly used herbal bath formulas for insomnia disorder will be examined. We believe that this review will offer evidence-based advice for the health care system, optimize treatment strategies for clinicians and identify promising herbal bath formulas for researchers.

## Author contributions

F Feng is the guarantor. F Feng conceived and designed this review. Q Wen and XH Dong contributed to drafting the protocol. Steve Tumilty, L Lan and LZ Liu were involved in critically revising the protocol. Q Wen and XH Dong will be responsible for the searching, screening and selecting studies. Q Wen, XH Dong, BH Yan, YB Chen, X Wang, and YG Zhang will perform data analyses and draft the manuscript. All authors will read and approve the final manuscript.

**Conceptualization:** Fen Feng.

**Funding acquisition:** Lizhou Liu.

**Methodology:** Yonggang Zhang.

**Software:** Bohua Yan, Yabing Chen.

**Writing – original draft:** Qiao Wen, Xiaohui Dong.

**Writing – review & editing:** Lei Lan, Lizhou Liu, Steve Tumilty, Fen Feng.

## Supplementary Material

Supplemental Digital Content
